# Supplementary material for: Nonlinear stimulus representations in neural circuits with approximate excitatory-inhibitory balance
Source: PLoS Comput Biol. 2020 Sep 18;16(9):e1008192. doi: 10.1371/journal.pcbi.1008192 (PMC7526938; doi:10.1371/journal.pcbi.1008192)
Supplement: S1 Fig — A–C) Same as Fig 3Bi–3Biii except that a conductance-based model was used for synapses. Synaptic currents from population a were measured by −ga(t)(V(t) − Ea). D–E) Same as A–B except “effective” synaptic currents were measured by Ia(t) = −ga(t)(V0 − Ea) where we chose V0 = −55mV, but results did not depend sensitively on the choice of V0. This defines a notion of effective balance and semi-balance in terms of a balance or semi-balance between the effective currents, instead of actual currents. Effective semi-balance and dominance of effective inhibition is an experimentally testable prediction of our model. (PDF) [file pcbi.1008192.s001.pdf]

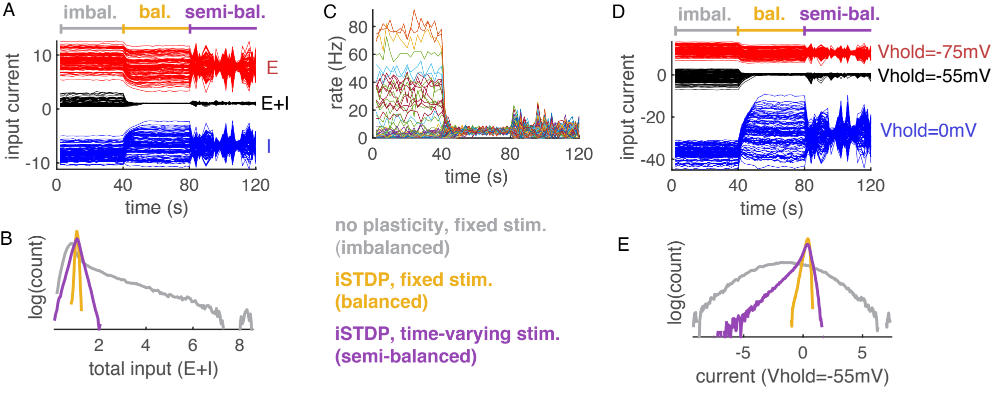
A
imbal.
bal.
semi-bal.
input current
10
0
-10
E
E+I
I
0
40
80
120
time (s)
B
log(count)
2
4
6
8
total input (E+I)
C
rate (Hz)
80
60
40
20
0
0
40
80
120
time (s)
no plasticity, fixed stim.
(imbalanced)
iSTDP, fixed stim.
(balanced)
iSTDP, time-varying stim.
(semi-balanced)
D
imbal.
bal.
semi-bal.
input current
0
-20
-40
Vhold=-75mV
Vhold=-55mV
Vhold=0mV
0
40
80
120
time (s)
E
log(count)
-5
0
5
current (Vhold=-55mV)
