# Supplementary figures and images for: Nonlinear stimulus representations in neural circuits with approximate excitatory-inhibitory balance
Source: PLoS Comput Biol. 2020 Sep 18;16(9):e1008192. doi: 10.1371/journal.pcbi.1008192 (PMC7526938; doi:10.1371/journal.pcbi.1008192)

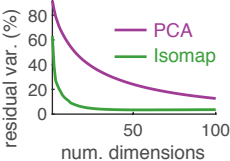

Supplement: S2 Fig — Same as Fig 3Ciii except IsoMap and PCA were applied to firing rates of layer 1 neurons from the model in Fig 4. (PDF) [file pcbi.1008192.s002.pdf]

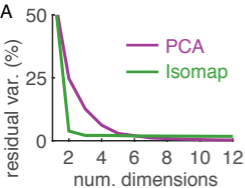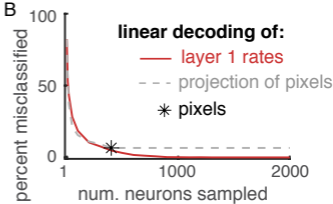

Supplement: S3 Fig — A) Same as Fig 3Ciii except the network was not trained by iSTDP. B) Same as Fig 4B except the network was not trained by iSTDP. (PDF) [file pcbi.1008192.s003.pdf]

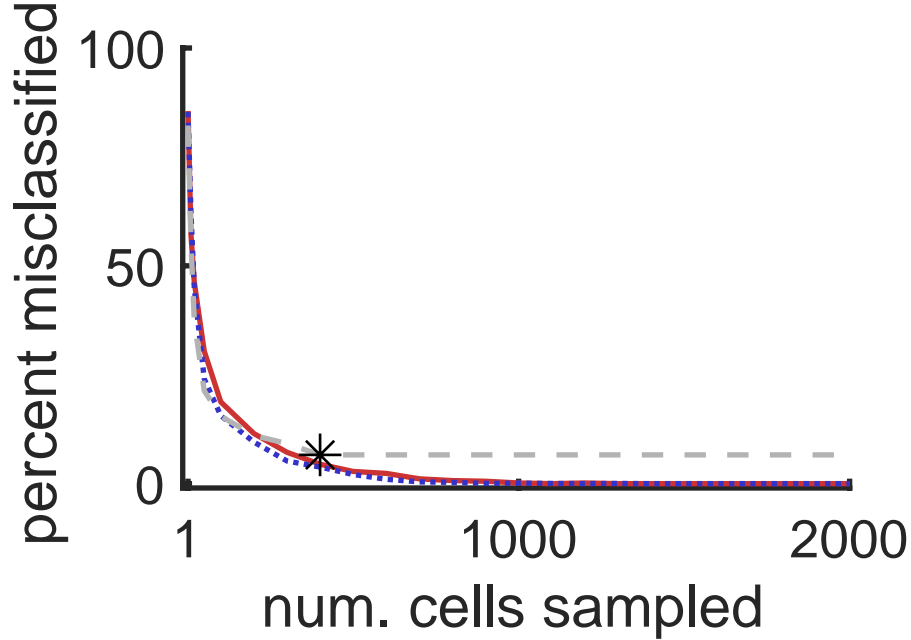

Supplement: S4 Fig — Same as Fig 4B except the dotted blue curve was added which represents the same as the red curve except the firing rate representation was replaced by a representation in which the raw pixels were projected randomly into 4000 dimensions, then passed through a rectified linear function. Specifically, the pixels were multiplied by a 400×n matrix of standard normal numbers (the same matrix for each digit) then passed through the function f(x) = [x]+ = max(x, 0). (PDF) [file pcbi.1008192.s004.pdf]
