# Supplementary material for: Nonlinear stimulus representations in neural circuits with approximate excitatory-inhibitory balance
Source: PLoS Comput Biol. 2020 Sep 18;16(9):e1008192. doi: 10.1371/journal.pcbi.1008192 (PMC7526938; doi:10.1371/journal.pcbi.1008192)
Supplement: Attachment — Submitted filename: Response1.pdf [file pcbi.1008192.s005.pdf]

# Response to Reviewers

We thank the reviewer for their helpful comments and corrections that have substantially improved the manuscript. Below, we respond to each of the reviewers' comments and describe changes to the manuscript in response to these comments. Reviewer's comments are in regular text, our responses are in boldfaced text, and text added to the manuscript is in red. Note that citation numbers in this document refer to the bibliography at the end of this document, not the main text.

## Reviewer 1

Comments:

1. The improvement on MNIST is nice, even though MNIST is a simple benchmark by modern standards. However, the results could be more convincing. Part of the reason is that we don't have a comparison to standard methods, and it's hard to do so because the authors are using a different setup (fewer images, lower-dimensional pixel space). A few suggestions:

   (a) Could the authors also report the performance of their approach applied to a random projection of the data into a space of the same dimensionality followed by a ReLU (a standard random one-hidden-layer network)? Even better would be to also show a network trained with backprop, which presumably would outperform both approaches.

   (b) The authors should also describe their "optimal linear readout" more carefully. It sounds like they're minimizing an l2 loss between a linear projection of the firing rates and the one-hot output. But this is not what is typically considered an "optimal" readout, namely a maximum-margin classifier (i.e. support vector machine). The authors should see if their results change if they use an SVM classifier.

   **We do not mean to suggest that the representation implemented by our network would outperform a backprop trained neural network or even a random layer of rectified linear units. More generally, we did not intend to claim that our network implements an especially good representation of MNIST digits, only that it is *better than linear*.**

   **The reviewer's suggestion to compare results to a random, rectified linear layer is a nice way to demonstrate this point. We made the comparison and, indeed, found that the random ReLu representation performed similarly to the representation implemented by our network. We added Supplementary Figure S9 to show this result.**

   **We considered the reviewer's suggestion to use a maximum-margin classifier, but this is classically only defined for binary classification problems in contrast to the multi-class MNIST problem we are considering. We did compute the maximum margin classifier between all *pairs* of digits and found that they are each pairwise separable in pixel space (which we reported in the paragraph below). We also pointed out that a support vector machine**

**or backprop trained neural net would be able to classify all of the digits accurately since zero misclassification errors on a training set of 2000 MNIST digits is a very easy task for a neural net or SVM.**

**We agree with the reviewer that it is not accurate to refer to our readout as "optimal" so we deleted the word "optimal" and instead described the classification as a "thresholded linear readout" which is a more accurate and precise description of what we did. We also added a more detailed description of the procedure, as well as a justification for choosing one-hot encoding and $\ell^2$ loss in Results and Discussion.**

**The modified and added text in the Results is:**

> We wondered if the nonlinearity of this representation imparted computational advantages over a linear representation. The 10 different digits (0-9) form ten clusters of points in the 4000-dimensional space of layer 1 excitatory neuron firing rates. Similarly, the raw images represent ten clusters of points in the 400-dimensional pixel space. Can these clusters of points be classified perfectly by thresholding a linear readout?
>
> To answer this question, we defined a linear readout of the 2000 firing rate vectors into 10 dimensions and trained the readout weights to be maximized at the dimension corresponding to the digit's label. Specifically, we defined a $10 \times 4000$ readout matrix, $W_r$ and a 10-dimensional readout vector, $\vec{x} = W_r \vec{r}_e$, where $\vec{r}_e$ is the $4000 \times 1$ vector of excitatory neuron firing rates in layer 1. We then minimized the $\ell^2$ loss function,
>
> $$L = \sum_{i=1}^{2000} \|\vec{x}_i - \hat{x}_i\|_2^2$$
>
> where $\vec{x}_i$ is the readout for MNIST digit $i = 1, \ldots, 2000$ and $\hat{x}_i$ is the one-hot encoding of the label ($\hat{x}_i$ is a $10 \times 1$ vector for which the $j$th element is equal to 1 when $j$ is the $i$th digit's label, and all other elements are zero). We chose a one-hot encoding because it allowed us to test whether digits could be classified by thresholding a linear readout. We chose an $\ell^2$ loss because it can be minimized explicitly without any dependence on hyperparameters.
>
> Using this procedure, we found that all 2000 digits were classified perfectly by thresholding the trained linear readout of firing rates.

**The added paragraph in Discussion is:**

> We showed that thresholding a linear readout perfectly classified 2000 MNIST digits encoded in firing rate space, but not pixel space. While optimal linear classification is well-defined for two classes, for example by maximum margin classifiers, there is not one universally optimal way to linearly classify data into several categories. We trained the readouts on a one-hot encoding of the labels using an $\ell^2$ loss. Other types of classifiers could lead to perfect classification in pixel space. For example, support vector machines and artificial neural networks trained with backpropagation perform extremely well on MNIST and could easily obtain perfect classification on a training set of 2000 digits. Also,

we found that each pair of digits is separable by a hyperplane in pixel space. Indeed, the binary separability of pairs of digits should be expected by Cover's Function Counting Theorem, which says that perfect binary classification of $m$ random points in $N$ dimensions is possible with high probability when $N$ is large and $m/N < 2$ [6]. Since there are about $m = 200$ images in each class (2000 digits with 10 classes) and the images live in $N = 400$ dimensions, we have and $m/N = 200/400 = 0.5$, implying that the images are well within the margin specified in Cover's Theorem. Our results should not be interpreted to imply that the firing rate representations implemented by our spiking networks are especially well-suited to solving MNIST, but rather that they are just one example of a random, sparse, non-linear representation, which are known to improve discriminability [1]. Indeed, repeating our analysis on a random rectified linear layer (representing an untrained, randomly initialized hidden unit) in place of our spiking network gives similar results (S9 Figure).

2. The mathematical notation would benefit from a re-read and could be cleaned up. As a few comments:

   - Line 95: $r_x$ should be bolded

     **Thank you for catching this typo. We fixed it and checked for other instances of the same typo.**

   - Line 98: average(.) and mean(.) are both used in the paper, use consistent standard terminology.

     **Thank you for catching this. We changed average(.) to mean(.).**

   - Both bold and arrow notation is used for vectors, stick with bold.

     **The use of two different vector notations was a deliberate choice used to distinguish between two different types of vectors. We use bold for mean-field vectors, such as $\mathbf{r} = [r_{e1}\ r_{e2}\ r_i]^T$, that represent averages over populations and have dimension equal to the number of populations. We use the arrow notation for $N$-dimensional vectors that have one element for each neuron. While it is perhaps somewhat awkward to use two different notational conventions in the same paper, it is difficult to think of a nicer notation that would distinguish between the 3-dimensional $\mathbf{r}$ and the $N$-dimensional $\vec{r}$ without introducing additional variable names that would become unwieldy.**

     **We kindly request that we can leave the notation as-is. However, your comment helped us realize that the notational convention was never explained in the text. To remedy this oversight, we added the following text in the place where the arrow notation is first used:**

     Note that we use the arrow notation, $\vec{I}$, for $N$-dimensional vectors to distinguish them from boldfaced mean-field vectors, like $\boldsymbol{I}$, that have dimensions equal to the number of populations. We apply the same notational convention to $\vec{r}$, $\vec{X}$, etc.

   - I'm not a fan of the "e1" and "e2" and "x1" and "x2" notation. Why not just 1 and 2 for the excitatory populations and x and y for the external populations? This one is not so crucial, more of an aesthetic point.

**We see merit in either approach. Our ongoing research deals with models having an arbitrary number of excitatory, inhibitory, and external populations. For such models, the approach suggested by reviewer would not work since "1" and "2" does not distinguish excitatory versus inhibition, and since "$x$" and "$y$" does not permit an arbitrary (i.e., parameterized) number of external populations. We also added Appendix S7 to the current manuscript, in response to a suggestion by Reviewer 3, and that appendix considers a model with two excitatory and two inhibitory populations, which would make it difficult to use notation like "1" and "2" for the excitatory population. To promote consistency of notation across our models and papers, we kindly request that we can stick with the current notation.**

Minor comments:

- In Figure 2, I wasn't able to understand the expression for $\beta = |E + I|/E$ at first because the brackets for the absolute value look the same as a capital I.

  **We replaced $|\cdot|$ with abs$(\cdot)$ to remedy this issue.**

- Theorem 2 of the S2 appendix seems to start with some missing characters.

  **We inserted the missing characters:** Suppose

- Line 743: "directions in which J only points weakly" – this is a bit confusing, since J is a matrix, not a vector.

  **We changed the text to:**

  directions, $\vec{v}$, in which $\|J\vec{v}\| \ll \|\vec{v}\|$

- Line 466: The statement that artificial neural networks often use sigmoids instead of ReLUs is a bit out of date – ReLUs are standard now.

  **In response to this comment and comments from another reviewer, we removed the paragraph in question.**

## Reviewer 2

Criticism and questions:

- The premise of this study is that Balance Networks entails a linear relation of the mean firing rates of different populations (the Balance Equations), thus cannot perform nonlinear operations. While the population averages keep a linear relation, the high dimensional activity is not necessarily linear. It is reasonable that computations in a balanced state are carried through higher statistics (e.g., local fluctuations) while keeping the mean activity steady.

  **This is an important point that was also brought up by another reviewer as well as some of our colleagues. We added the following paragraph to the Discussion to address it. The paragraph refers to the new S7 Appendix, which was added in response to a suggestion by Reviewer 3).**

Classical balanced networks are balanced at the population level, but not necessarily at the level of individual neurons (no detailed balance). While such networks can only perform linear computations at the level of population averages, they can perform nonlinear computations at the level of single neurons and their firing rate fluctuations [24, 14, 13]. Cortical circuits do appear to perform nonlinear computations at the population level. For example, population responses to high-contrast visual stimuli add sub-linearly, which can be captured by SSNs [22] and semi-balanced networks (see S7 Appendix).

- The authors ignore the fluctuations altogether in the paper. With their normalization (line 489), the fluctuations are expected to be O(1). It is not apparent to me if there are further restrictions on the connectivity when multiple populations are concerned, and what will be the stability criteria. While the authors acknowledge not studying stability (line 433), its absence is a significant drawback of this report.

  **We agree that studying fluctuations and dynamics is a fruitful direction of research, but it is outside the scope of this initial study of the semi-balanced state. We added some details about stability and dynamics (including stability conditions for multiple populations, as requested by the reviewer) to this paragraph in the Discussion:**

  One limitation of our approach is that it focused on fixed point rates and did not consider their stability or the dynamics around fixed points. Indeed, fluctuations of firing rates and total synaptic inputs are $\mathcal{O}(1)$ under the scaling of synaptic weights that we used. When a solution to Eq. (4) exists, it represents a fixed point of Eq. (1) in the $\overline{JK} \to \infty$ limit. The fixed point is stable when all eigenvalues of the Jacobian matrix of Eq. (1) evaluated at the fixed point have negative real part. Previous work shows that balanced networks can exhibit spontaneous transitions between attractor states [16] which can be formed by iSTDP [25, 17]. Attractor states in those studies maintained strictly positive firing rates across populations, keeping the networks in the classical balanced state. This raises the question of whether similar attractors could arise in which some populations are silenced by excess inhibition, putting them in a semi-balanced state. Tools for studying these states, and for studying stability and dynamics more generally, could potentially be developed from the mathematical theory of threshold-linear networks [9, 26, 8, 7].

- The nonlinear computations presented are equivalent to a nonlinear expansion of the input space into higher dimensions. This procedure has been shown to improve the network classification performance on otherwise linearly-inseparable data [Babadi and Sompolinsky, Neuron, 2014].

  **As noted below (in response to one of the "Minor issues and comments"), we added a citation to Babadi and Sompolinsky [1] and a paragraph about its relationship with our results.**

  It is not clear to me why and to what extent the balance is required for that. Are the authors assuming balance as a given state, or are they suggesting that balance is beneficial? I think a simple network of nonlinear units will do just that without balance. Is the balance a way to

get an effective firing-rate based unit out of spiking neurons? Is this a way to reduce readout fluctuations [Deneve and Machens, Nat. Neuro. 2016] and provide robustness to external noise? I think the authors should address the role and importance of (semi)-balance in their results.

**We did not mean to suggest that balance is required for nonlinear representations or nonlinear expansion. Figures 3 and 4 are only meant to investigate the properties of the nonlinearities produced by detailed semi-balanced networks trained by iSTDP. Networks that are not trained by iSTDP can implement nonlinear representations, but are imbalanced at the level of single neurons (detailed imbalance). Our results show that a form of detailed balance can be achieved while preserving nonlinear representations. We do not mean to argue that balance is beneficial in any specific way in this context. To clarify these points, we added Supplementary Figure S8 in which we reproduce the nonlinearities of Figures 3 and 4 without iSTDP and we added the following paragraph to the Discussion:**

> We showed that networks with iSTDP achieve detailed semi-balance and produce nonlinear representations at the level of individual neurons (Figs. 3 and 4). However, we do not mean to suggest that iSTDP or balance is responsible for the presence of nonlinear representations or the linear separability of MNIST images in rate space. iSTDP is needed for achieving detailed semi-balance, not nonlinear representations. Indeed, repeating simulations from Figs. 3 and 4 without iSTDP gives similar results (see S8 Figure). However, networks without iSTDP are imbalanced at the resolution of individual neurons (detailed imbalance, see Fig. 3B, gray). In summary, our results show that networks with iSTDP can produce a form of detailed balance (detailed semi-balance) while still implementing nonlinear representations.

Minor issues and comments

- In the model of the 2D input (Figure 3, and paragraph starting line 265). What is the role of the external population x? is it to simulate background noise? Following van Vreeswijk and Sompolinsky, I believe the model should work without the noisy input, with only a DC input. The strength of the input from the noisy external population should provide evidence to the robustness to noise.

  **This is an interesting observation. We added a paragraph to explain our choice to use noisy, spike-based synaptic input as follows:**

  > Since we are primarily interested in the encoding of the perturbation, $\vec{Z}$, we could have replaced the spike-based, Poisson synaptic input from the external population with a time-constant, DC input to each neuron as in previous work [23]. We chose to keep the spike-based input to add biological realism and to demonstrate the the encoding of $\vec{Z}$ is robust to the Poisson noise induced by the background spike-based input. A more biologically realistic model might encode $\vec{Z}$ in the spike times themselves instead of using an additive perturbation.

- In the paragraph starting at line 370, the authors ask what the number of neurons needed to achieve separability is. It seems to be more of an observation on the data instead of on the model. As a comparison, they could use the theoretical results by Cover on the dimensions needed to allow linear classification (P=2N, where N is the number of neurons, and P is the number of points).

  **This is an excellent suggestion. Note, however, that Cover's Theorem applies to binary classification, and we are considering classification of MNIST digits, which is multi-class. However, we did check whether all *pairs* of digits were separable by a hyperplane in pixel space and found that they were separable. This finding is consistent with Cover's Theorem, as we described in this text added to the Discussion section:**

  > Also, we found that each pair of digits is separable by a hyperplane in pixel space. Indeed, the binary separability of pairs of digits should be expected by Cover's Function Counting Theorem, which says that perfect binary classification of $m$ random points in $N$ dimensions is possible with high probability when $N$ is large and $m/N < 2$ [6]. Since there are about $m = 200$ images in each class (2000 digits with 10 classes) and the images live in $N = 400$ dimensions, we have and $m/N = 200/400 = 0.5$, implying that the images are well within the margin specified in Cover's Theorem.

- The 2-layers model (Fig 4) seems analogous to sparse expansion with feedforward inhibition (Babadi and Sompolinsky, 2014). The authors should relate their models to the known results.

  **We added the following paragraph:**

  > We found that the nonlinearities implemented by semi-balanced networks can improve the separability of MNIST digit representations. Previous work shows that high-dimensional, sparse representations can improve decoding [1]. This could help to understand our empirical results since representations in the semi-balanced state are sparse in the sense that some proportion of neurons are silent for any given stimulus.

  **We also cited [1] in the following text which was added to address another reviewer's comment:**

  > Our results should not be interpreted to imply that the firing rate representations implemented by our spiking networks are especially well-suited to solving MNIST, but rather that they are just one example of a random, sparse, non-linear representation, which are known to improve discriminability [1].

- line 459: Recurrent spiking neural networks are notoriously difficult to train.... Training spiking networks has been done both for Deep Networks [e.g., Zenke and Ganguli, 2017] and in recurrent networks [Nicola and Clopath, Nature 2017]

**We did not mean to suggest that recurrent spiking networks had never been trained, only that the complex relationship between connectivity, neural dynamics, and firing rates can make it difficult. To clarify, we changed the sentence to:**

> The relationship between connectivity and firing rates in recurrent spiking networks can be mathematically difficult to derive, which can make it difficult to derive gradient based methods for training recurrent spiking networks (though some studies have succeeded, see for example [18, 12]).

**We excluded the reference to (Zenke and Ganguli, 2017) here because it does not seem that their networks were recurrent, so the citation would not be consistent with our sentence.**

- Line 466: Artificial recurrent neural networks for machine learning often use sigmoidal activation functions instead of the rectified linear activations typically used in feedforward networks because the unboundedness of rectified linear units make recurrent networks susceptible to instabilities and large activations. RNN with ReLU units can be stabilized even if it is unbound, and in particular, with balance [Kadmon and Sompolinsky, PRX, 2015]. Stabilization is achieved by adequately normalizing the weight variance near the transition to chaos. The same also applies to Deep Networks with ReLU [Poole et al. 2016, arXiv:1606.05340] and with tanh activation [Pennington et al. 2017, arXiv:1711.04735]

  **In response to these comments and comments from the another reviewer, we removed the paragraph in question.**

## Reviewer 3

Major comments

1. The critique of the classical balanced state found in the introduction seems a bit overblown.

   - l.49-50: Satisfying constraints on parameters in the standard balanced network is very easy (only a few inequalities on parameters), and there is no known model in which satisfying these constraints has been found to be difficult. Furthermore, homeostatic mechanisms such as the iSTDP mechanism proposed in this manuscript is an easy way out in case initial parameters do not satisfy the inequalities. All in all, this issue does not seem at all to be a 'critical shortcoming'.

     **In the commonly studied case of two populations (one excitatory and one inhibitory) there are just two inequalities that need to be satisfied. However, each additional population introduces another inequality to be satisfied. In general, $n$ inequalities divide parameter space into $\sim 2^n$ disjoint regions and only one of those regions represents a valid, positive firing rate vector. The specific parameterization of the network connectivity and external input determines how easy it is to find parameters within this region, so it is difficult**

**to make precise and general statements about how easy positive firing rates can be achieved.**

**However, note that the positivity of the balanced firing rate solutions depends on both the connectivity matrix and the external input vector, so a connectivity matrix that gives positive balanced rates for one input might give negative rates for another. Indeed, we proved that any connectivity matrix satisfying Dale's law admits some positive external inputs that produce negative balanced rate solutions (so any network admits some excitatory stimuli that violate balance). Since a given fixed network would receive multiple stimuli over time, we interpret this to mean that any network is susceptible to inputs that break the classical balanced state.**

**As suggested by the reviewer, a network with one external input value trained by iSTDP will typically converge to a connectivity structure that achieves its positive target rates. However, a network with iSTDP that receives *multiple external stimuli* will not necessarily converge to a state that achieves positive balanced rate solutions for all such stimuli. Indeed, no network could achieve balance for all stimuli (as noted above). At the level of detailed balance studied in Fig. 3, this fact is demonstrated by the large negative synaptic inputs and quenched firing rates observed in some neurons in response to some stimuli (different neurons are silenced by different stimuli). In our ongoing work, we have observed the same thing at the population level: Even with just a few populations and a few different external stimuli, iSTDP often finds a steady-state connectivity matrix in which the classical balanced rate equations would predict negative rates for some populations (hence they are silenced in the semi-balanced state). To summarize, iSTDP only solves the issue of negative balanced rates if we restrict ourselves to a single external input stimulus (or if we switch the stimuli more slowly than the iSTDP learning rate, which is not realistic).**

**Regarding the reviewer's comment that "there is no known model in which satisfying these constraints has been found to be difficult," we know of at least two cases. In Supplementary Section S.2 of [19], balanced networks with 6, 8, and 10 populations (3, 4, and 5 excitatory and inhibitory populations) were considered. The connectivity matrices were purposefully parameterized in a way that promotes positivity of the balanced firing rates by adding a constant along the diagonal of the connectivity matrix. Even with this parameterization, only about 50% of the randomly sampled parameter values produced positive rates. The others were discarded because the balanced network theory considered in that paper could not handle them. The semi-balanced theory developed in the current manuscript would have been able to resolve this issue. Secondly, [15] considered balanced networks with heterogeneous degree distributions. They required sufficiently strong adaptation current to avoid the presence of net-negative synaptic input, which is mathematically equivalent to the negative predicted firing rates from the balance equations in our analysis (see their Equation 5 and surrounding discussion). Taking all of this into consideration, we feel that the issue of positive firing**

**rates is significant. However, to avoid possibly overstating the issue, we removed the word "critical" from "critical shortcoming." We also clarified the issue by rewriting the paragraph in question as follows:**

> Secondly, parameters in balanced network models must be chosen so that the firing rates predicted by balanced network theory are non-negative. In the widely studied case of one excitatory and one inhibitory population, parameters for network connectivity and external input must satisfy only two inequalities to achieve positive predicted rates [23, 21]. However, strictly positive predicted rates can be more difficult to achieve in networks with several populations such as multiple neuron subtypes, neural assemblies, or tuning preferences [15, 19]. This difficulty occurs because the proportion of parameter space for which predicted rates are non-negative becomes exponentially small with an increasing number of populations. Moreover, a given network architecture might produce a balanced state for some stimuli, but not others. Indeed, we show that for any network architecture satisfying Dale's law, there are infinitely many excitatory stimuli for which balanced network theory predicts negative rates, implying that any network structure admits stimuli that break the classical balanced state.

- l.61: It is not obvious why the results in ref.[19] would be incompatible with the standard balanced state picture - in that paper the authors mention that inhibitory conductances are much larger than excitatory conductances in the awake state, but this is not necessarily incompatible with a balance of currents.

  **We agree that the fact that Haider et al [10] only report conductances, not currents, makes it difficult to determine whether their results are consistent with the balanced state. We removed the sentence in question from the Introduction and removed a similar sentence from Results. We re-wrote a paragraph in the Discussion to give a more careful comparison of our results to those in [10] and to more carefully describe what our theory predicts and doesn't predict for synaptic currents and conductances:**

> The semi-balanced state is defined by an excess of inhibition without a corresponding excess of excitation. This is at first glance consistent with evidence that inhibition dominates cortical responses in awake animals [10]. However, it should be noted that synaptic conductances, not currents, were reported in and they only reported conductances relative to their peaks, not raw conductances [10]. It is therefore difficult to draw a direct relationship of the results in [10] to our results on balance or semi-balance. In addition, we found that the dominance of inhibitory synaptic currents is reduced when shunting inhibition is accounted for (Fig. 2Cii and S5 Figure). Hence, due to shunting inhibition, our model does not necessarily predict a strong excess of inhibitory currents in the semi-balanced state. A more precise prediction of our model is that stimuli will silence a subset of neurons through shunting inhibition (Fig. 2Ci,ii), consistent with evidence that visual inputs evoke shunting inhibition in cat visual cortex [3]. In addition, if synaptic currents are measured under voltage clamp with the potential clamped sufficiently far between the excitatory and

inhibitory reversal potentials, we predict a skewed distribution of currents with a heavier tail of hyperpolarizing versus depolarizing currents (S5 Figure, as in Fig. 3Biii purple). These predictions should be tested more directly using *in vivo* recordings.

2. The authors remain quite vague in the paper about what types of non-linearities are observed in cortex, and whether their model can reproduce these specific types of non-linearities (as opposed to a generic ability of exhibiting non-linear responses). For instance, in visual cortex one typically sees linear summation of two stimuli at low contrast, but sublinear summation at high contrast (see ref. 30). Can the model exhibit these non-linearities?

   **We demonstrated an XOR linearity (Figure 1F) because XOR is a classical example of a function that cannot be computed by a linear layer, but we did not consider additional nonlinearities at the population level. Per the reviewers request, we added Supplementary Section S7 Appendix, which demonstrates that a 4-population model of two receptive field locations can produce the nonlinearity cited by the reviewer. We also added the following paragraph to the Discussion section to describe these new results:**

   > We demonstrated that semi-balanced networks can implement a continuous XOR nonlinearity at the population level (Fig. 1F) and detailed semi-balanced networks implement more intricate nonlinearities at the resolution of single neurons (Fig. 3C,D), but we did not consider additional types of nonlinearities. Recordings show that visual cortical neurons exhibit a nonlinearity in which low-contrast visual stimuli sum linearly while high-contrast stimuli sum sub-linearly, a phenomenon that can be reproduced by supralinear stabilized networks (SSNs) [22]. In S7 Appendix we show that this type of nonlinearity can also be captured by a simple semi-balanced network that obeys Eq. (4). Future work should more completely explore the types of nonlinearities that can be expressed by solutions to Eq. (4).

3. In the section on semi-balance in networks with heterogeneous inputs and iSTDP is somewhat confusing. In particular, while the role of iSTDP in producing detailed (semi) balance is clear, its role in producing non-linear representations is not.

   The classic balanced network model features linearity of the AVERAGE firing rates as a function of input, but single neuron responses can be quite non-linear (van Vreeswjik and Sompolinsky, in Methods and Models in Neurophysics, 2005, Elsevier).

   **This is an important point that was also brought up by another reviewer as well as some of our colleagues. We added the following paragraph to the Discussion to address it:**

   > Classical balanced networks are balanced at the population level, but not at the level of individual neurons (no detailed balance). While such networks can only perform linear computations at the level of population averages, they can perform nonlinear computations at the level of single neurons and their firing rate fluctuations [24, 14, 13]. Cortical circuits do appear to perform nonlinear computations at the population level. For example, population responses to high-contrast visual

stimuli add sub-linearly, which can be captured by SSNs [22] and semi-balanced networks (see S7 Appendix).

Also, strongly heterogeneous inputs will themselves push neurons outside of their linear range in the absence of iSTDP. The question therefore arises whether similar non-linear representations could arise in networks with no iSTDP. To check this, the authors could repeat the analysis described in panels C in networks with no iSTDP.

**We did not mean to suggest that balance is required for nonlinear representations or nonlinear expansion. Figure 3 and 4 are only meant to investigate the properties of the nonlinearities produced by detailed semi-balanced networks trained by iSTDP. Networks that are not trained by iSTDP can implement nonlinear representations, but are imbalanced at the level of single neurons (detailed imbalance). Our results show that a form of detailed balance can be achieved while preserving nonlinear representations. We do not mean to argue that balance is beneficial in any specific way in this context.**

**To clarify these points, as suggested by the reviewer, we added Supplementary Figure S8 in which we reproduce the nonlinearities of Figures 3 and 4 without iSTDP and we added the following paragraph to the Discussion:**

> We showed that networks with iSTDP achieve detailed semi-balance and produce nonlinear representations at the level of individual neurons (Figs. 3 and 4). However, we do not mean to suggest that iSTDP or balance is responsible for the presence of nonlinear representations or the linear separability of MNIST images in rate space. iSTDP is needed for achieving detailed semi-balance, not nonlinear representations. Indeed, repeating simulations from Figs. 3 and 4 without iSTDP gives similar results (see S8 Figure). However, networks without iSTDP are imbalanced at the resolution of individual neurons (detailed imbalance, see Fig. 3B, gray). In summary, our results show that networks with iSTDP can produce a form of detailed balance (detailed semi-balance) while still implementing nonlinear representations.

Another issue with iSTDP is that distributions of firing rates recorded in cortex are typically very wide, and much closer to the no iSTDP case than the case with iSTDP. It has been shown that such broad distributions are an automatic by-product of the random connectivity in balanced networks (e.g. Roxin et al 2011). From this point of view, the standard balanced network model seems more realistic that the one with iSTDP where the distribution of rates is narrow (unless one puts by hand a broad distribution of target firing rates).

**This is an interesting observation. Note that the width of the firing rate distribution is sensitive to the parameters defining the time-dependent perturbations given to the network. It is possible that naturalistic perturbations could lead to more realistically distributed firing rates in the presence of iSTDP and/or more unrealistic (too broadly distributed) firing rates in the absence of iSTDP. We added the following paragraph to address this potential issue:**

Firing rates in the detailed semi-balanced state are not very broadly distributed (Fig. 3Bii, last 40s), which is inconsistent some cortical recordings. Note that the broadness of the firing rate distribution is partly a function of the magnitude of the perturbation strengths, $\sigma_1$ and $\sigma_2$. Also, all of our perturbations lie on a two-dimensional plane, so they could potentially be balanced more effectively by iSTDP than higher dimensional perturbations. Finally, our iSTDP rule used the same target rate for all neurons, which may not be realistic. Stronger perturbations, higher-dimensional perturbations, and variability in target rates, among other factors, could lead to broader firing rate distributions in the detailed semi-balanced state. The width of firing rate distributions for naturalistic stimuli should be considered in future work, but is outside the scope of this study.

Finally, the authors should provide some intuition why the distribution of total inputs is left skewed. Is this because of the specific functional form of the equation in line 544, which produces large jumps in the case of excess excitation but not in the case of excess inhibition ($x_j^E$ can grow to really large values but cannot go below zero)?

**There are two ways of giving an intuition for this result. The first is to more precisely generalize the notion of semi-balance to the "detailed" situation (semi-balance at each neuron) and explain how the skewed distribution is predicted by detailed semi-balance. The second way, as suggested by the reviewer, is to understand how the mechanics of the iSTDP rule produce excess inhibition, but not excitation. We added paragraphs to explain both approaches to intution. First, we added a paragraph to more precisely characterize the notion of excess inhibition under semi-balance at the population level:**

In other words, populations in the semi-balanced state can receive $\mathcal{O}(\overline{JK})$ net-inhibitory input, but if their input is net-excitatory, it must be $\mathcal{O}(1)$. Hence, the semi-balanced state is characterized by excess inhibition, but not excess excitation, to some neural populations. In contrast, the balanced state requires net-input to be $\mathcal{O}(1)$ regardless of whether it is net-excitatory or net-inhibitory, hence no excess excitation or inhibition. Note that firing rates remain $\mathcal{O}(1)$ in both the balanced and semi-balanced states.

**We next added a paragraph generalizing this notion of excess inhibition without excess excitation to the "detailed" level:**

Specifically, generalizing the definitions of population-level balance and semi-balance above, detailed balance is defined by requiring that the net synaptic input to all neurons is $\mathcal{O}(1)$. Detailed semi-balanced only requires neurons' input to be $\mathcal{O}(1)$ when it is net-excitatory. Net-inhibitory input to some neurons will be $\mathcal{O}(\overline{JK})$ in the detailed semi-balanced state. As such, the distribution of total synaptic input to neurons in the semi-balanced state will be left-skewed, indicating strong inhibition to some neurons, but no comparably strong excitation.

**We then explained how this prediction of detailed semi-balance is consistent with the left-skewed distribution in Fig. 3:**

> Of course, real cortical circuits receive time-varying stimuli. To simulate time-varying stimuli, we randomly selected new values of $\sigma_1$ and $\sigma_2$ every 2s (Fig. 3Bi-ii last 40s). This change lead to some neurons receiving excess inhibition in response to some stimuli, but neurons did not receive correspondingly strong excess excitation (Fig. 3Bi, black curves last 40s) resulting in a left-skewed distributions of synaptic inputs (Fig. 3Biii purple). These results are consistent with a detailed semi-balanced state, which is characterized by excess inhibition to some neurons, but a lack of similarly strong excitation. These results show that detailed semi-balance, but not detailed balance, is naturally achieved in circuits with iSTDP and time-varying stimuli.

**Finally, we explained the mechanics of how this left-skewed distribution (and therefore detailed semi-balance) is achieved by iSTDP:**

> To gain a better intuition for why the distribution in (Fig. 3Biii, purple) is left-skewed, consider the network with iSTDP and time-varying stimuli. iSTDP changes weights in a way that encourages all excitatory firing rates to be close to a target rate [25] (we used a target rate of 5 Hz). In the presence of a stimulus that varies faster than the iSTDP learning rate, the network cannot achieve the target rates for every neuron at every stimulus. However, the network is pushed strongly away from states with large, net-excitatory input to some neurons because those states produce large firing rates that are very far from the target rates. On the other hand, the network is not pushed as strongly away from states with large net-inhibitory input to some neurons because those states produce firing rates of zero for those neurons, which is not so far from the target rates.

4. The authors show that, when inputs evolve dynamically, the learned representation is nonlinear. The authors should comment on the fact that, depending on the history of the stimuli presented over time, the same stimulus can have completely different representations. This seems to have major implications for sensory encoding.

   **This is a good observation. We added the following sentence:**

   > It is worth noting that, due to the presence of plasticity, the same stimulus presented at two different points in time might not have the same firing rate representation.

5. In the section: "Nonlinear representations in semi-balanced networks improve computations", they show that linear separability of images improves when they are fed into a network of E-I neurons. As in the previous section, it is unclear what role iSTDP plays exactly. As in point 3 above, I believe they should compare results obtained with and without iSTDP.

   **We did not mean to suggest that iSTDP was necessary for nonlinear representations or linear separability of MNIST digit representations. iSTDP was only**

**used to achieve detailed balance and semi-balance, not to achieve nonlinear representations. We added Supplementary Figure S8 in which we show that networks without iSTDP also implement nonlinear representations and achieve linear separability of MNIST digits. And we added the following paragraph to the Discussion to clarify this issue:**

> We showed that networks with iSTDP achieve detailed semi-balance and produce nonlinear representations at the level of individual neurons (Fig. 3 and 4). However, we do not mean to suggest that iSTDP or balance is responsible for the presence of nonlinear representations or the linear separability of MNIST images in rate space. iSTDP is needed for achieving detailed semi-balance, not nonlinear representations. Indeed, repeating simulations from Figs. 3 and 4 without iSTDP gives similar results (see S8 Figure). However, networks without iSTDP are imbalanced at the resolution of individual neurons (detailed imbalance, see Fig. 3B, gray). In summary, our results show that networks with iSTDP can produce a form of detailed balance (detailed semi-balance) while still implementing nonlinear representations.

Minor comments:

- It could be a good idea to mention that the simplest possible semi-balanced network has just two populations, one silent E population and an active I population.

  **This is a nice observation. We added the following paragraph:**

  > It is worth noting that the simplest possible semi-balanced network has one inhibitory population and one excitatory population with the excitatory population silenced by the inhibitory population. This would arise when a condition for the positivity of firing rates in a two-population balanced network is violated [23, 21].

- In line 109 (page 3), ref[8]: Technically, the van Vreeswijk-Sompolinsky papers did not use spiking networks. To my knowledge, a linear relationship between firing rates and external inputs in networks of spiking neurons was first derived by Brunel in 2000.

  **Thank you for catching this. We changed it to spiking networks and binary networks. Thank you also for pointing out the derivation of the linear-balanced regime in Brunel's 2000 paper, which we had not previously noticed among all of the other notable results in that paper. We added a citation to the paper at this place. We additionally noted that knowledge of an f-I curve need not be assumed [2]. The sentence now reads:**

  > While Eq. (1) is a heuristic approximation to spiking networks, Eq. (3) can be derived for spiking networks and binary networks in the limit of large $\overline{JK}$ without appealing Eq. (1) and even without specifying an f-I curve at all [23, 5, 2].

- In line 110 (page 3). N not defined.

  **We added the following text at the line noted by the reviewer:**

  where $N$ is the number of neurons in the recurrent network

- Figure 2B, How was the ISN property tested?

  **We added the following text to the Methods to explain our procedure:**

  > To distinguish between ISN and non-ISN regimes, we computed the Jacobian matrix of the network, checked that all eigenvalues had negative real part (verifying that the fixed point was stable), then checked the eigenvalues of the excitatory sub-matrix of the Jacobian (the matrix with the inhibitory column and row removed). The eigenvalues of the full matrix always had negative real part (the fixed point was always stable). If the eigenvalues of the excitatory sub-matrix had positive real part, we classified the network as an ISN at those parameter values, otherwise it was classified as non-ISN.

- In the methods, it would be good to provide some justification of why this specific single neuron model was chosen, and of the choice of parameters:

  **We added:**

  > We chose the adaptive EIF neuron model because it is simple and efficient to simulate while also being biologically realistic [4, 11].

  - l.498, synaptic weights of order $1/\sqrt{N}$: Shouldn't they be of order $1/\sqrt{K}$? Of course, the choice would be important only if the authors analyzed how network behavior changes as a function of $K$ or $N$, something which is not done in this paper.

    **Since our networks are densely connected ($p_{ab}$ fixed, so $K \sim N$), scaling by $\sqrt{N}$ is equivalent to scaling by $\sqrt{K}$. The convention of scaling by $\sqrt{N}$ has been used in some balanced network studies after it was shown that densely connected balanced networks produce asynchronous spiking activity [20]. We chose to use $N$ as a scaling factor because a precise value of $K$ is a more complicated number to define and interpret, especially in multi-population models because neurons in different populations may have different in-degrees. We added the following sentence to clarify this issue:**

    > Note that some balanced network studies scale weights by $\sqrt{K}$ instead of $\sqrt{N}$. Since we keep connection probabilities fixed, $K \sim N$, so scaling by $\sqrt{N}$ is equivalent to scaling by $\sqrt{K}$.

  - l.508-515: The description of how synaptic strengths are computed in the conductance-based version is confusing - I believe the current-based weights should be divided by the driving force to get the conductance-based weights, not the opposite. This confusion is present both in the first and the last sentence of the paragraph.

    **Thank you for catching this mistake. This paragraph was originally adapted from a paragraph in another of our papers in which we describe the normalization in the opposite direction: from conductance to current, which explains the mistake. We switched from "multiplied" to "divided" to accurately reflect the scaling that we performed in the code, which is the correct scaling, as the reviewer suggested.**

– The description of the connectivity from pixels to layer 1 is also confusing and does not seem to match the ratio of number of neurons to pixels. With 400 pixels, the indicated procedure would work if there were 40,000 neurons, but there are only 4,000 excitatory neurons. Shouldn't 100 be replaced by 10? Also, the value of $\sigma$ is not specified.

**Thank you for catching this error. We changed 100 to 10 where necessary. We also indicated the value of $\sigma = 20$mV.**

## References


[1] Baktash Babadi and Haim Sompolinsky. Sparseness and expansion in sensory representations. *Neuron*, 83(5):1213–1226, 2014.

[2] C Baker, C Ebsch, I Lampl, and R Rosenbaum. Correlated states in balanced neuronal networks. *Phys Rev E*, 99(5):052414, 2019.

[3] Lyle J Borg-Graham, Cyril Monier, and Yves Fregnac. Visual input evokes transient and strong shunting inhibition in visual cortical neurons. *Nature*, 393(6683):369, 1998.

[4] Romain Brette and Wulfram Gerstner. Adaptive exponential integrate-and-fire model as an effective description of neuronal activity. *J Neurophysiol*, 94(5):3637–3642, 2005.

[5] Nicolas Brunel. Dynamics of sparsely connected networks of excitatory and inhibitory spiking neurons. *J Comput Neurosci*, 8(3):183–208, 2000.

[6] Thomas M Cover. Geometrical and statistical properties of systems of linear inequalities with applications in pattern recognition. *IEEE Transactions on Electronic Computers*, 3(EC-14):326–334, 1965.

[7] Carina Curto, Jesse Geneson, and Katherine Morrison. Fixed points of competitive threshold-linear networks. *Neural Comput*, 31(1):94–155, 2019.

[8] Carina Curto and Katherine Morrison. Pattern completion in symmetric threshold-linear networks. *Neural Comput*, 28(12):2825–2852, 2016.

[9] Richard HR Hahnloser and H Sebastian Seung. Permitted and forbidden sets in symmetric threshold-linear networks. In *Advances in neural information processing systems*, pages 217–223, 2001.

[10] Bilal Haider, Michael Häusser, and Matteo Carandini. Inhibition dominates sensory responses in the awake cortex. *Nature*, 493(7430):97–100, jan 2013.

[11] Renaud Jolivet, Felix Schürmann, Thomas K Berger, Richard Naud, Wulfram Gerstner, and Arnd Roth. The quantitative single-neuron modeling competition. *Biological cybernetics*, 99(4-5):417, 2008.

[12] Christopher M Kim and Carson C Chow. Learning recurrent dynamics in spiking networks. *Elife*, 7:e37124, 2018.

[13] Guillaume Lajoie, Kevin K Lin, and Eric Shea-Brown. Chaos and reliability in balanced spiking networks with temporal drive. *Phys Rev E*, 87(5):052901, 2013.

[14] Guillaume Lajoie, Kevin K Lin, Jean-Philippe Thivierge, and Eric Shea-Brown. Encoding in balanced networks: Revisiting spike patterns and chaos in stimulus-driven systems. *PLoS Comp Bio*, 12(12):e1005258, 2016.

[15] Itamar D Landau, Robert Egger, Vincent J Dercksen, Marcel Oberlaender, and Haim Sompolinsky. The impact of structural heterogeneity on excitation-inhibition balance in cortical networks. *Neuron*, 92(5):1106–1121, 2016.

[16] Ashok Litwin-Kumar and Brent Doiron. Slow dynamics and high variability in balanced cortical networks with clustered connections. *Nat Neurosci*, 15(11):1498, 2012.

[17] Ashok Litwin-Kumar and Brent Doiron. Formation and maintenance of neuronal assemblies through synaptic plasticity. *Nat Comm*, 5:5319, 2014.

[18] Wilten Nicola and Claudia Clopath. Supervised learning in spiking neural networks with force training. *Nature communications*, 8(1):1–15, 2017.

[19] R Pyle and R Rosenbaum. Highly connected neurons spike less frequently in balanced networks. *Phys Rev E*, 93(4):040302, 2016.

[20] A Renart, J de La Rocha, P Bartho, L Hollender, N Parga, A Reyes, and KD Harris. The Asynchronous State in Cortical Circuits. *Science*, 327(5965):587–590, 2010.

[21] R Rosenbaum and B Doiron. Balanced networks of spiking neurons with spatially dependent recurrent connections. *Phys Rev X*, 4(2):021039, 2014.

[22] Daniel B Rubin, Stephen D Van Hooser, and Kenneth D Miller. The stabilized supralinear network : A unifying circuit motif underlying multi-input integration in sensory cortex. *Neuron*, 85(2):1–51, 2015.

[23] C van Vreeswijk and H Sompolinsky. Chaotic balanced state in a model of cortical circuits. *Neural Comput*, 10(6):1321–1371, 1998.

[24] C van Vreeswijk and H Sompolinsky. Methods and models in neurophysics course 9: Irregular activity in large networks of neurons. *Les Houches*, 80:341–406, 2005.

[25] T P Vogels, H Sprekeler, F Zenke, C Clopath, and W Gerstner. Inhibitory plasticity balances excitation and inhibition in sensory pathways and memory networks. *Science*, 334(6062):1569–73, dec 2011.

[26] Xiaohui Xie, Richard HR Hahnloser, and H Sebastian Seung. Selectively grouping neurons in recurrent networks of lateral inhibition. *Neural Comput*, 14(11):2627–2646, 2002.
